# The anti-adiposity effect of bitter melon seed oil is solely attributed to its fatty acid components

**DOI:** 10.1186/s12944-017-0578-3

**Published:** 2017-09-29

**Authors:** Gou-Chun Chen, Wen-Hung Chen, Kuo-Tang Tseng, Pei-Min Chao

**Affiliations:** 1 Department of Nutrition, China Medical University, Taichung, 404 Taiwan; 2 Aquavan Technology Co., Ltd, Taipei City, Taiwan

**Keywords:** Bitter melon seed oil, Plasma leptin, Sirt1 mRNA, Thermogenic protein, Mice C57BL/6 J

## Abstract

**Background:**

Obesity is the leading chronic disease affecting people of all ages. The objective of this study was to optimize composition of a bitter melon seed oil (BMSO) product to maximize its anti-adiposity effect.

**Methods:**

Bleaching oil, saponifiables and non-saponifiables were prepared from BMSO, with α-eleostearic acid (α-ESA) content in BMSO maintained in bleaching oil and saponifiables. C57BL/6 J mice were allocated into five groups (*n* = 10/group) to receive diet C [30% soybean oil (SBO)], BM [25% SBO + 5% BMSO], BMS, BMNS or BMD. For the three latter diets, saponifiables (hydrolyzed fatty acids from BMSO), non-saponifiables (excluding fatty acids from BMSO) or bleaching oil (excluding pigments from BMSO), respectively, were added in amount equivalent to their content in 5% BMSO and SBO was added to bring total fat to 30%. After 14 wk., indices associated with adiposity and safety, as well as lipid metabolic signaling in white adipose tissue (WAT), were measured.

**Results:**

The body fat percentage of mice in group BM, BMS, BMNS, and BMD were 90 ± 26, 76 ± 21, 115 ± 30 and 95 ± 17% of that in group C. Based on body fat percentage and plasma leptin concentrations, an anti-adiposity effect was evident in groups BM, BMS and BMD (greatest effect in BMS). Histologically, inguinal fat had smaller adipocytes in groups BM, BMS and BMD (*P* < 0.05), but not in group BMNS, relative to group C. There were no differences among groups in blood pressure or heart rate. Moreover, *Sirt1* mRNA levels in inguinal fat were significantly greater in groups BM, BMS and BMD than group C.

**Conclusion:**

We concluded that the anti-adiposity function of BMSO was solely attributed to the fatty acid fraction, with the free fatty acid form having the greatest effect.

**Electronic supplementary material:**

The online version of this article (10.1186/s12944-017-0578-3) contains supplementary material, which is available to authorized users.

## Background

Obesity, a complex metabolic disorder, is the leading chronic disease affecting people of all ages. Effective and safe agents that can be used as adjuncts to decrease body fat deposition are urgently needed. We reported that bitter melon seed oil (BMSO) was more potent than soybean oil (SBO) in attenuating body fat accumulation via cAMP-activated protein kinase (PKA) and leptin activation in white adipose tissue (WAT) in diet-induced obese mice [1]. In oils extracted from seed of 10 varieties of bitter melon (*Momordica charantia*), α-eleostearic acid (α-ESA; *cis*-9, *trans*-11, *trans*-13- isomer of conjugated linolenic acid) comprised 30–60% of total fatty acids [2].

Using the 3 T3-L1 preadipocyte cell line, we determined that α-ESA was far less potent than its non-conjugated counterpart, α-linolenic acid, or other common unsaturated C18 fatty acids in stimulating adipocyte differentiation [3]. This effect was partly ascribed to the apoptotic effect of α-ESA on proliferating [3, 4] and differentiating preadipocytes [3]. Conjugated linolenic acid (CLNA) is a collective term for a group of positional and geometric isomers of linolenic acid with at least two conjugated double bonds. Punicic acid (*cis*-9,*trans*-11,*cis*-13 isomer of CLNA), catalpic acid (*trans*-9,*trans*-11,*cis*-13 isomer of CLNA), calendic acid (*trans*- 8,*trans*-10,*cis*-12 isomer of CLNA), and a CLNA mixture of *cis*-9,*trans*-11,*cis*-15 and *cis*-9,*trans*-13,*cis*-15 isomers, had anti-obesity potential both in vivo and in vitro [5–8].

In addition to α-ESA, other fat-soluble phytochemicals, including phytosterols (β-sitosterol and stigmasterol), pigments (lutein and lycopene) and phytol have been identified in the whole fruit [9] or seed coat [10] of bitter melon. These compounds have favorable effect on lipid metabolism, including upregulating fatty acid β-oxidation via peroxisome proliferator-activated receptor α (PPARα) activation [11], sirtuin 1 (SIRT1) activation [12, 13], or modulation of microRNA [14]. We speculated that these compounds, despite their low concentrations, may act synergistically with α-ESA for anti-obesity.

The objective was to optimize composition of a BMSO product to maximize the anti-adiposity effect; therefore, components with potential for synergy with α-ESA were explored. An animal feeding trial was conducted to compare anti-adiposity effects among BMSO and saponifiables, non-saponifiables and bleaching oil from BMSO, with SBO alone as a control. Results should be useful for development of safe and effective functional food products.

## Methods

### Preparation of BMSO

BMSO was prepared by solvent extraction [15] with details as described [1]. Bitter melon seed (supplied by Hualien District Agricultural Research and Extension Station, Hualien, Taiwan) was powdered, dissolved in 10 volumes of n-hexane and agitated overnight at room temperature. After filtration through Whatman filter paper (No 1), residue was re-extracted as above, and filtrates were combined and evaporated under reduced pressure and used as BMSO. The yield was 25 g from 100 g of bitter melon seed.

### Preparation of bleaching BMSO

Bleaching BMSO was prepared as described [16], with slight modifications. The BMSO was dissolved in n-hexane (1:1, w:v), mixed with 3% activated carbon (0.325 mm) for 1 h at room temperature, and centrifuged (15,000 × g for 10 min) to collect the decolorized oil supernatant. The yield of bleaching oil was 89 g from 100 g BMSO.

### Preparation of saponifiables and non-saponifiables of BMSO

Following Hsu et al. [9], BMSO was saponified by dissolving it in 10-fold volume of 3.6 N KOH/methanol and incubating it at room temperature overnight. Then, solvent was evaporated and residue partitioned in ethyl acetate (EA) and distilled water (3–5 times). The EA and water fractions were collected for preparation of non-saponifiable and saponifiable fractions, respectively, of BMSO. Aqueous fractions were further acidified with 5 N H_2_SO_4_ to reach pH 2 and then extracted (twice) with an equal volume of EA. The upper phase (EA extract) was collected and washed with water until the aqueous phase was pH 7. Thereafter, organic solvent was evaporated to yield saponifiables of BMSO (83% yield). In addition, after saponification, the EA fraction was collected and evaporated to yield non-saponifiables (1% yield).

### Thin-layer chromatography

Aliquots of BMSO, bleaching oil, saponifiables and non-saponifiables were separately dissolved in chloroform (10 mg/mL). Thin-layer chromatography on a silica gel 60 plate developed by a 9/1 (*v*/v) mixture of petroleum ether/80% acetone was used to confirm hydrolysis of BMSO into free fatty acids. To visualize development, plates were immersed in 10% sulfuric acid and baked at 100 °C for 1 min.

### ‬UV spectrometry

An α-ESA standard (quoted purity >98%) was purchased from Cayman (Ann Arbor, MI, USA). The BMSO, bleaching oil, saponifiables, non-saponifiables and α-ESA standard were individually dissolved in n-hexane (7 μg/mL) and UV spectrometry (U-2000, Hitachi, Tokyo, Japan) used to measure absorbances between 200 and 300 nm (1-nm resolution).

### Animals and diets

Male C57BL/6 JNarl mice were purchased from the National Laboratory Animal Center of the National Applied Research Laboratories, Taipei, Taiwan. At 6 wk. of age, mice were randomly allocated into five groups, i.e. C, BM, BMS, BMNS, and BMD (*n* = 10 per group), and fed one of the test diets which were modified from AIN-93G [17] (Table 1).The 30% dietary fat composed of SBO alone (C), 25% SBO + 5% BMSO (BM), 25.06% SBO + 4.94% saponifiables (BMS), 29.94% SBO + 0.06% non-saponifiables (BMNS), 25.55% SBO+ 4.45% bleaching oil (BMD). In this context, C served as a control and BM served as a positive control. For BMS, BMNS and BMD diet, saponifiables, non-saponifiables or bleaching oil, respectively, were added in amounts equivalent to their content in 5% BMSO and total fat was increased to 30% by addition of SBO. All mice were kept in a room maintained at 23 ± 2 °C on a controlled 12-h light:dark cycle with ad libitum access to food and tap water. Diets were stored in sealed containers filled with nitrogen, and fresh food was supplied every other day. Body weight was recorded weekly. After 14 wk. of dietary treatment, food was withheld overnight and mice were killed by carbon dioxide asphyxiation. Adipose tissues (retroperitoneal, epididymal, and inguinal fat) were excised and weighed. Blood was collected in EDTA tubes and plasma was separated by centrifugation (3000×*g* for 10 min at 4 °C). Plasma leptin concentrations were measured using an enzyme-linked immunosorbent assay (R&D, Minneapolis, MN, USA).

**Table 1:** Composition (g/100 g feed) of test diets used in this study

Component	C	BM	Diet BMS	BMNS	BMD
Corn starch	16	16	16	16	16
Casein	26	26	26	26	26
Cellulose	6.1	6.1	6.1	6.1	6.1
Sucrose	16	16	16	16	16
Soybean oil	30	25	25.06	29.94	25.55
BMSO	–	5			
Saponifiables			4.94		
Non-saponifiables				0.06	
bleaching oil					4.45
AIN- 93 Mineral mix	4.2	4.2	4.2	4.2	4.2
AIN- 93 Vitamin mix	1.2	1.2	1.2	1.2	1.2
DL-Cystine	0.3	0.3	0.3	0.3	0.3
Choline bitartrate	0.2	0.2	0.2	0.2	0.2

C, soybean oil-based high-fat diet; BM, soybean oil-based high-fat diet containing BMSO; BMS, soybean oil-based high-fat diet containing saponifiables of BMSO; BMNS, soybean oil-based high-fat diet containing non-saponifiables of BMSO; BMD, soybean oil-based high-fat diet containing bleached BMSO

### Adipocyte cell diameter

Fixed inguinal fat was dehydrated through a graded ethanol series, embedded in paraffin, cut into 5-μm sections, and examined under a light microscope (OLYMPUS I × 71, Tokyo, Japan) equipped with a SPOT RT color-2000 digital camera (Diagnostic Instruments, Sterling Heights, MI, USA) to obtain images; adipocyte cell diameter was estimated with Adiposoft software (ImageJ; National Institutes of Health, Bethesda, MD, USA).

### Blood pressure and heart rate

After 13 wk on the diets, diastolic and systolic blood pressures and heart rate were measured using a tail-cuff system (MK-2000ST, Muromachi Kikai Co., Ltd., Tokyo, Japan) that uses a photoelectric sensor to detect blood flow in the tail. Mice were acclimated to the procedure for 7 consecutive days prior to blood pressure and heart rate recordings on day 8. For each mouse, at least 1 set of 10 measurements with 9 or more successful readings, was obtained.

### RNA isolation and mRNA detection

Total RNA was extracted from inguinal fat using TRIZOL reagent (Invitrogen, San Diego, CA, USA) according to the manufacturer’s instructions. The quality of the extracted RNA was confirmed by a value of 2 for the 28S: 18S ribosomal RNA ratio after ethidium bromide staining. Total RNA (1 μg) was reverse-transcribed into first-strand cDNA using 200 units of MMLV-RT in a total volume of 20 μL. For real-time PCR, a SYBR system (Applied Biosystems, Foster, CA, USA) and primers designed in our laboratory (Additional file 1), were used. Amplification using 40 cycles of two steps (95 °C for 15 s and 60 °C for 1 min) was performed on an ABI Prism 7900HT sequence detection system (Foster City, CA, USA). Quantitative values were obtained from the threshold cycle value (Ct), the point at which a significant increase of fluorescence is first detected. Calculation of the relative mRNA concentration was made using the 2^-ΔΔCt^-method, with GAPDH as a reference gene.

### Immunoblotting

Inguinal fat was homogenized in RIPA buffer containing 1% protease inhibitor cocktail and 1% phosphatase inhibitor cocktail (Sigma) and samples (30 μg of protein) were subjected to electrophoresis on 10% SDS gels, transferred to a polyvinylidene fluoride-plus transfer membrane (NEN Life Science, Boston, MA, USA), and immunoblotted. Primary antibodies (diluted 1:1000 in PBS) were rabbit antibodies against human UCP1, AMPK catalytic subunit α, phospho-AMPKα (Thr172), ACC, phospho-ACC (Ser 79) and GAPDH, whereas HRP-labeled donkey anti-rabbit IgG (Amersham International, Buckinghamshire, UK) at a dilution of 1:5000 in PBS was the secondary antibody. Bound antibodies were detected using an enhanced chemiluminescence Western blotting kit (Amersham International) and images were quantified by densitometric analysis using a Multimage Light Cabinet (Alpha Innotech, San Leandro, CA, USA).

### Statistical analyses

Data were expressed as mean ± SD. Comparisons among groups were done with 1-way ANOVA and Duncan’s multiple range test. If variances were not homogeneous, data were log-transformed prior to analysis. The General Linear Model (SAS, SAS Institute, Cary, NC, USA) was used for statistical analyses and differences were considered significant at *P* < 0.05.

## Results

### Separation and properties of saponifiables, non-saponifiables and bleaching oil

**Fig. 1:**
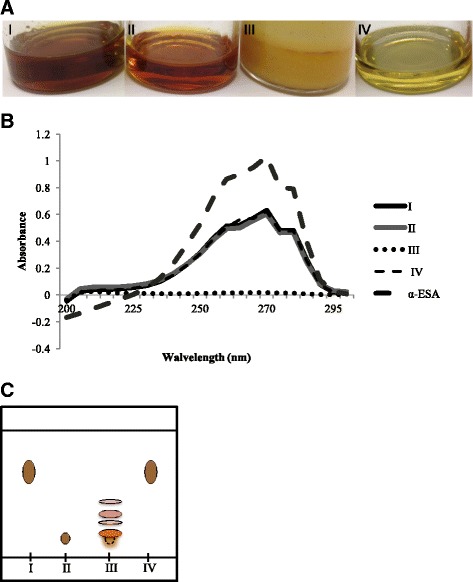
Appearance (**a**), UV spectrum (**b**), and thin-layer chromatography results (**c**) of BMSO and its fractions. I, BMSO; II, saponifiables; III, non- saponifiables; IV, bleached BMSO

The appearance of BMSO, saponifiables, non-saponifiables and bleaching oil are shown (Fig. 1a). The BMSO was dark brown, but became light yellow after decolorization (Fig. 1a, I versus IV), suggesting pigments were efficiently removed by adsorption with activated carbon. The color of saponifiables (Fig. 1a, II) was intermediate between BMSO and bleaching oil, indicating partial removal of pigments. However, compared to non-saponifiables (Fig. 1a, III), saponifiables were much clearer. Measurement of α-ESA can be done with UV-VIS spectrometry [18] which peaks at 270 nm. Wavelength scans from 200 to 300 nm of all four products, as well as α-ESA standard, are shown (Fig. 1b ). At a consistent concentration (7 μg/mL), OD_270_ was 0.63, 0.60, 0.02, 0.62, and 1.03 for BMSO, saponifiables, non-saponifiables, bleaching oil and α-ESA standard, respectively, indicating an equal amount of α-ESA (~60% of oil) in BMSO, saponifiables, and bleaching oil, while with an absence in non-saponifiables. Thin-layer chromatography (Fig. 1c) confirmed efficient hydrolysis of BMSO, as there was no residue of triglycerides in saponifiables. In this normal phase chromatography, triglyceride (nonpolar) moves faster than free fatty acids (FFA). Non-saponifiables contained trace FFA contamination and polar compounds (migrated between triglyceride and FFA).

### Adiposity indices

**Fig. 2:**
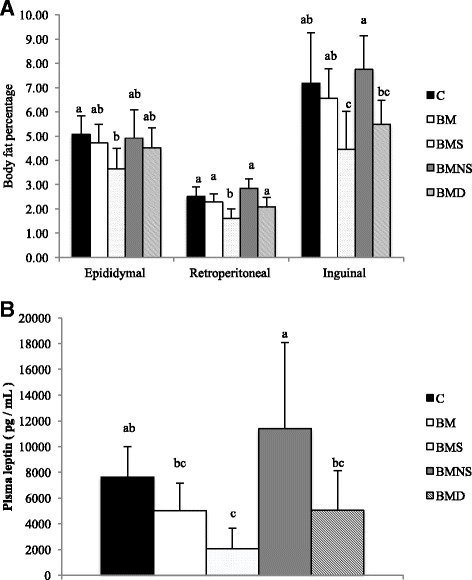
Body fat percentage (**a**) and plasma leptin concentrations (**b**) of mice fed SBO-based high-fat diets containing various fractions of BMSO for 14 wk. C, soybean oil-based high-fat diet; BM, soybean oil-based high-fat diet containing BMSO; BMS, soybean oil-based high-fat diet containing saponifiables of BMSO; BMNS, soybean oil-based high-fat diet containing non-saponifiables of BMSO; BMD, soybean oil-based high-fat diet containing bleached BMSO. Results are mean ± SD (*n* = 10) ^a-c^Means without a common letter differed (*P* < 0.05)

**Fig. 3:**
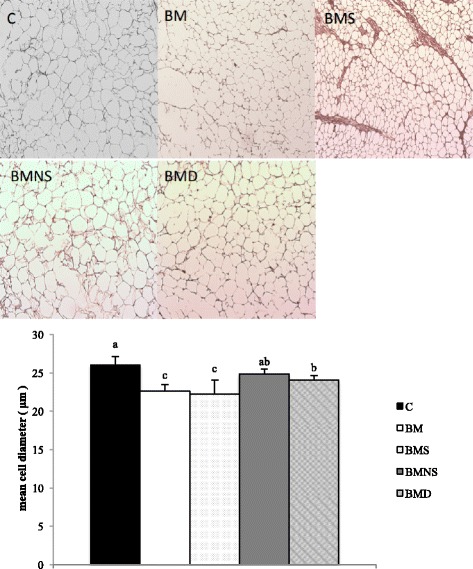
Adipocyte size of mice fed SBO-based high-fat diets containing various fractions of BMSO for 14 wk. C, soybean oil-based high-fat diet; BM, soybean oil-based high-fat diet containing BMSO; BMS, soybean oil-based high-fat diet containing saponifiables of BMSO; BMNS, soybean oil-based high-fat diet containing non-saponifiables of BMSO; BMD, soybean oil-based high-fat diet containing bleached BMSO. Results are mean ± SD (*n* = 10 mice/group). ^a-c^ Means without a common letter differed (*P* < 0.05)

During the 14-wk intervention period, energy intake did not differ among groups (data not shown). Based on body fat percentage (Fig. 2a) and plasma leptin concentrations (Fig. 2b), as indicators of total body fat mass, mice fed saponifiables (group BMS) had the lowest values among groups (significantly less than groups C or BMNS). Compared to group C, there were varying degrees of anti-adiposity effects for BMSO (group BM), saponifiables (group BMS) and bleaching oil (group BMD), but not for non-saponifiables (group BMNS). The body fat percentage of mice in group BM, BMS, BMNS, and BMD were 90 ± 26, 76 ± 21, 115 ± 30 and 95 ± 17% of that in group C. Consistent with these findings, cell diameter in inguinal fat (Fig. 3) was significantly reduced in group BMD, with further reductions in groups BMS and BM, but not at all in group BMNS, relative to group C. In general, based on these indices, the anti-adiposity effect was greatest for saponifiables, followed by BMSO (irrespective of decolorization). However, the anti-adiposity effect totally disappeared when fatty acids were removed from BMSO.

### Blood pressure and heart rate

As BMSO is effective in attenuating body fat accumulation through mechanisms associated with sympathetic activation, i.e. β-adrenergic receptor/PKA signaling in the WAT [1], we measured blood pressure and heart rate at the end of intervention. There were no differences among groups for either heart rate (473.88 ± 25.53 beats/min) or blood pressure (126.20 ± 8.84 and 78.12 ± 4.87 mmHg for systolic and diastolic blood pressure, respectively). Furthermore, these values were within the normal range [19].

### Thermogenic proteins and signaling in WAT

**Fig. 4:**
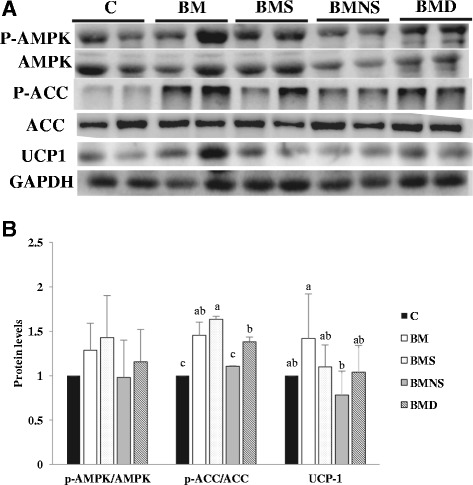
Phosphorylation levels of AMPK and ACC protein and thermogenic protein UCP1 in inguinal fat of mice fed SBO-based high-fat diets containing various fractions of BMSO for 14 wk. Representative immunoblot (**a**). Signals were quantified by image analysis and results expressed as phosphorylated/total protein ratio of AMPK and ACC, as well as ratio of UCP1/GAPDH (**b**). C, soybean oil-based high-fat diet; BM, soybean oil-based high-fat diet containing BMSO; BMS, soybean oil-based high-fat diet containing saponifiables of BMSO; BMNS, soybean oil-based high-fat diet containing non-saponifiables of BMSO; BMD, soybean oil-based high-fat diet containing bleached BMSO. Results are mean ± SD (*n* = 10 mice/group) ^a,b^ Means without a common letter differed (*P* < 0.05).

We had reported that a high dose (15%) of BMSO increased thermogenesis in WAT [1, 20, 21]. Here, we measured proteins associated with thermogenesis and energy homeostasis in WAT of mice subjected to low-dose BMSO and its fractions (Fig. 4a). At a lower dose of BMSO (5%), induction of WAT browning was not as obvious as that of high dose; however, for UCP1 protein in inguinal fat, there was a significant difference between groups BM and BMNS, with intermediate values for other groups. AMP-activated protein kinase (AMPK) serves as an energy switch, which phosphorylates and inactivates lipogenic enzymes such as acetyl-CoA carboxylase (ACC) [22]. In accordance with a slightly higher phosphorylation levels of AMPKα in groups BM, BMS and BMD than groups C and BMNS, phosphorylation levels of ACC in groups BM, BMS and BMD were significantly higher than group C and BMNS (Fig. 4b).

**Fig. 5:**
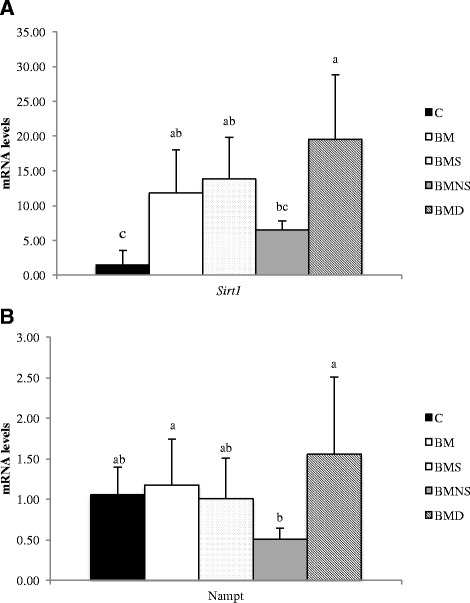
Levels of mRNA for Sirt1 (**a**) and Nampt (**b**) in inguinal fat of mice fed SBO-based high-fat diets containing various fractions of BMSO for 14 wk. C, soybean oil-based high-fat diet; BM, soybean oil-based high-fat diet containing BMSO; BMS, soybean oil-based high-fat diet containing saponifiables of BMSO; BMNS, soybean oil-based high-fat diet containing non-saponifiables of BMSO; BMD, soybean oil-based high-fat diet containing bleached BMSO. Results are mean ± SD (*n* = 10 mice/group). ^a-c^Means without a common letter differed (*P* < 0.05)

We previously reported that α-ESA activated SIRT1, through increased mRNA levels and activity of nicotinamide phosphoribosyltransferase (NAMPT), a rate-limiting enzyme for NAD+ salvage synthesis [23] in a hepatocyte cell line [2]. SIRT1, as a NAD^+^-dependent deacetylase, has been implicated as a master controller that contributes to favorable metabolic effects associated with caloric restriction. The mRNA levels of *Sirt1* in inguinal fat in group BMD were significantly higher than groups BMNS and C, with intermediate values for groups BM and BMS (Fig. 5a). The mRNA levels of *Nampt* in groups BMD and BM were significantly higher than group BMNS, with groups BMS and C intermediate (Fig. 5b).

## Discussion

The anti-adipogenic effect of α-ESA, demonstrated in 3 T3-L1 cell cultures, is regarded as the main contributor to the anti-adiposity function of BMSO in vivo, although there other components may contribute synergistically [9, 10, 24]. Therefore, saponifiables (mainly comprised of hydrolysed fatty acids from BMSO), non-saponifiables (excluding fatty acids from BMSO) and bleaching oil (excluding pigments from BMSO) were prepared and tested on diet-induced obese mice for anti-adiposity function. Based on diet composition, if α-ESA was the sole functional component, the extent of body fat reduction would have been the same for groups BM, BMS and BMD. However, if there were active components present in non-saponifiables, efficacy would have been compromised in groups BMS or BMD relative to group BM. In the present study, there was clear evidence that anti-adiposity components were present in the fatty acid fraction. Non-fatty acid components, including lutein, lycopene, phytol, phytosterols [9, 10] or other triterpenoids [24], although present in bitter melon and bitter melon seed, may be in insufficient concentrations to exert synergistic effects on BMSO-mediated anti-adiposity function.

The most remarkable anti-adiposity effect occurred in group BMS. Though the possibility that some unknown ingredients in BMSO (perhaps in non-saponifiables) block the BMS-mediated anti-adiposity could not be excluded, we believe this was ascribed to greater intestinal bioavailability of FFA compared to the esterified form. In studies of fish oil supplements, absorption rate of eicosapentaenoic acid (EPA) + docosahexaenoic acid (DHA) was usually greatest for the FFA form relative to the triglyceride form, and lowest for the EE form. This was attributed to esterified forms requiring hydrolysis by pancreatic enzymes (secreted in response to fat intake) prior to being absorbed, whereas FFA do not require hydrolysis [25]. In addition, EPA + DHA in FFA form was superior to the EE form in reducing blood triglyceride concentrations [26]. However, marketing commercial BMSO products in FFA form has inherent challenges. Given the highly oxidizable nature of FFA and therefore the propensity to rapidly become rancid, products will need greater stability to have an extended shelf life.

We previously demonstrated BMSO activates β-adrenergic receptor/PKA signaling in WAT [1], thus raising safety concerns, since side effects of ephedrine (central nervous system stimulant) such as insomnia, worries, hypertension, and palpitation are well known. However, there was no increase in either blood pressure or heartbeat rate for mice subjected to this low dose of BMSO or its fractions. We reported BMSO increased tyrosine hydroxylase (TH) protein concentrations in WAT and that TH was responsible for catecholamine (i.e., adrenaline and noradrenaline) synthesis [21]. Therefore, we speculated BMSO increased concentrations of catecholamine in local WAT, which activated PKA signaling (by autocrine or paracrine mechanisms), thus contributing to increased lipolysis and thermogenesis.

Though evidence of WAT browning in this study was not as prominent as our previous reports (using high-dose BMSO [1, 20, 21]), plausible metabolic benefits of BMSO or α-ESA on WAT were expected. In inguinal fat, groups BM, BMS and BMD had significantly higher *Sirt1* mRNA levels and slightly higher AMPK activation than groups C and BMNS. It is noteworthy that SIRT1 acted as a novel upstream regulator of LKB1/AMPK signaling in the protective effect of polyphenols against high glucose-induced lipid accumulation in hepatocytes [27]. By deacetylating LKB1, SIRT1 influences its nuclear/cytoplasmic localization, binding to STE-related adaptor and activation of AMPK [28]. Sequential phosphorylation and deacetylation by AMPK and SIRT1 activates transcriptional coactivator peroxisome proliferator- activated receptor gamma coactivator-1α, a positive modulator of peroxisome proliferator-activated receptor α activity and mitochondrial biogenesis [29, 30], and prevents translocation of sterol regulatory element-binding protein 1c, a lipogenic transcription factor, into the nucleus [31, 32].

Based on overexpression or RNA interference, it has been clearly demonstrated that SIRT1 is a negative modulator of adipogenesis in 3 T3-L1 preadipocytes [33]. Meanwhile, it is believed that SIRT1 activation promotes fat mobilization in adipocytes through peroxisome proliferator-activated receptor γ (PPARγ) repression. Using ChIP assays, SIRT1 and PPARγ were demonstrated to bind to the same DNA sequences, suggesting that SIRT1 acted as a co-repressor of PPARγ [33]. The anti-adipogenic effect of AMPK was expected; based on its role in energy production and adipocyte differentiation, it is regarded as an energy-consuming process prohibited by AMPK activation [34]. In addition, via p38 MAPK, AMPK phosphorylates PPARγ, thus inhibiting its transcriptional activity and thereby blocking adipocyte differentiation [35, 36]. In accordance with this, a botanical supplement for weight management, Xanthigen, with punicic acid and fucoxanthin (from brown seaweed) as major components, had anti-adipogenic effects in 3 T3-L1 by up-regulating the SIRT1 and AMPK signaling pathway accompanied with downregulation of PPARγ [37].

In addition to α-ESA, many isomers of CLNA have anti-obesity potential. Punicic acid from pomegranate seed or genetically modified rapeseed oil decreased fat mass in mice with upregulated carnitine palmitoyltransferase activity in liver and brown adipose tissue [5, 38]. Catalpic acid from catalpa seed decreased abdominal fat accumulation, along with upregulated adipose PPARα in diet-induced obese and db/db mice [6]. Furthermore, calendic acid in its EE form was reported to reduce body fat in ICR mice though with low efficacy compared to conjugated linoleic acid (CLA), which has anti-adiposity function been extensively investigated and sold on market for weight loss [7]. Luciferase transactivation assay identified a mixture of CLNA isomers (*cis*-9,*trans*-11,*cis*-15 and *cis*-9,*trans*-13,*cis*-15) activated PPARα, but not PPARγ, and reduced triglyceride contents in 3 T3-L1 adipocytes along with increased expression of lipolytic enzymes [8]. Among these CLNA, only α-ESA and punicic acid are present in edible foods.

In contrast to many drugs and therapies which have been limited by side effects, research and development for functional foods or nutraceuticals holds a great potential for the anti-obesity market. Using a proteomic approach combined with histological evidence, we have shown WAT from BMSO-fed mice with features of caveolae reduction, ROS increase, tissue remodeling/repair, mitochondria uncoupling, actin cytoskeleton stabilization, and inflammation increase [20]. These features were very similar to the WAT of mice subject to CLA [39]. Though α-ESA and CLA both are PPARα activators [40] which enhance lipid catabolism, α-ESA and CLA seem to have unique effects on adipocytes since both reduce body fat in a PPARα-independent manner [21]. The underlying mechanisms for BMSO or α-ESA-mediated anti-adiposity function were attributed to (pre)adipocyte apoptosis and PKA activation [1, 20], and these effects persisted even with PPARα being ablated [21]. Commercial CLA product is chemically synthesized from base-catalyzed n6-PUFA-rich oil, while α-ESA or BMSO possesses the advantage of a natural source. Of course, the function awaits to be validated in human studies which may provide an opportunity for industries wishing to launch a new effective and safe product.

## Conclusion

We concluded that the anti-adiposity function of BMSO was solely attributed to its fatty acid fraction and that the FFA form was more effective than the triglyceride form. In the context of producing and marketing food, α-ESA may be used as an efficacy index for materials selectivity and quality control during processing. Therefore, these results should assist food processors to develop safe and effective functional food products.

## Additional file

Additional file 1: Gene names and sequences of PCR primers. (PDF 27 kb)

## Abbreviations

ACC: Acetyl-CoA carboxylase
AMPK: AMP-activated protein kinase
BMSO: Bitter melon seed oil
CLA: Conjugated linoleic acid
CLNA: Conjugated linolenic acid
DHA: Docosahexaenoic acid
EE: Ethyl ester
EPA: Eicosapentaenoic acid
FFA: Free fatty acid
GAPDH: Glyceraldehyde 3-phosphate dehydrogenase
NAMPT: Nicotinamide phosphoribosyltransferase
PKA: cAMP-activated protein kinase
PPARα: Peroxisome proliferator-activated receptor α
PPARγ: Peroxisome proliferator-activated receptor γ
SIRT1: Sirtuin 1
TH: Tyrosine hydroxylase
UCP1: Uncoupling protein 1
WAT: White adipose tissue
α-ESA: α-eleostearic acid
