# Supplementary material for: The anti-adiposity effect of bitter melon seed oil is solely attributed to its fatty acid components
Source: Lipids Health Dis. 2017 Sep 29;16:186. doi: 10.1186/s12944-017-0578-3 (PMC5622538; doi:10.1186/s12944-017-0578-3)
Supplement: Additional file 1: — Gene names and sequences of PCR primers. (PDF 27 kb) [file 12944_2017_578_MOESM1_ESM.pdf]

Additional files 1. Gene names and sequences of PCR primers.

Gene	Encoding protein	Accession number	Primer
Nampt	nicotinamide phosphoribosyl-transferase	NM_021524	F: AGCAG CAGAG CACAG TACCA<br>R: GCTAT CGCTG ACCAC AGACA
Sirt1	sirtuin 1	NM_019812	F: CCGCG GATAG GTCCA TATAC TT<br>R: CGCTT TGGTG GTTCT GAAAG G
